# Hollow Iron Oxide Nanospheres Obtained through a Combination of Atomic Layer Deposition and Electrospraying Technologies

**DOI:** 10.3390/nano12183180

**Published:** 2022-09-13

**Authors:** Paulina Márquez, Cristian Patiño Vidal, Alejandro Pereira, Leonardo Vivas, Juan Luis Palma, Carol López de Dicastillo, Juan Escrig

**Affiliations:** 1 School of Engineering, Central University of Chile, Santiago 8330601, Chile; 2 Center for the Development of Nanoscience and Nanotechnology (CEDENNA), Santiago 9170124, Chile; 3 Packaging Innovation Center (LABEN), University of Santiago de Chile (USACH), Santiago 9170201, Chile; 4 Departament of Sciences, Faculty of Liberal Arts, Adolfo Ibañez University, Santiago 7941169, Chile; 5 Department of Physics, University of Santiago de Chile (USACH), Santiago 9170124, Chile; 6 Packaging Laboratory, Institute of Agrochemistry and Food Technology (IATA-CSIC), 46980 Paterna, Spain

**Keywords:** magnetic properties, hollow nanospheres, atomic layer deposition, hematite (Fe_2_O_3_), magnetite (Fe_3_O_4_), poly(vinylpyrrolidone) (PVP)

## Abstract

In the present study, we report on the successful synthesis of hollow iron oxide nanospheres. The hollow Fe_3_O_4_ nanospheres were synthesized following a four-step procedure: electrospraying spherical PVP particles, coating these particles with alumina (Al_2_O_3_) and hematite (Fe_2_O_3_) through atomic layer deposition and, finally, a thermal reduction process to degrade the polymer (PVP) and convert hematite (Fe_2_O_3_) into magnetite (Fe_3_O_4_). A structural analysis using X-ray diffraction (XRD) confirmed the effectiveness of the thermal reduction process. A morphological analysis confirmed that the four-step procedure allowed for the obtainment of hollow iron oxide nanospheres, even though the reduction process caused a contraction in the diameter of the particles of almost 300 nm, but did not affect the thickness of the walls of the hollow spheres that remained at approximately 15 nm. Magnetic properties of the hollow iron oxide nanospheres enable their use in applications where the agglomeration of magnetic nanostructures in liquid media is commonly not allowed, such as in drug encapsulation and delivery.

## 1. Introduction

Ferrites have been intensively studied due to their interesting magnetic and photocatalytic properties and their low toxicity. In fact, they have been intensively used in magnetic memories, high-frequency devices and information storage systems, among others. In recent years, different groups have investigated nanometric ferrites because, on this length scale, ferrites exhibit extraordinary magnetic properties compared to their macroscopic counterpart. For example, magnetite (Fe_3_O_4_) has been extensively explored for its interesting nanoscale applications in drug delivery, gas sensors, catalytic activities and as an antibacterial agent [1,2,3,4,5,6]. Along with magnetite, other forms of iron oxides can appear altering their oxidation states, for example, hematite (α-Fe_2_O_3_), β-Fe_2_O_3_, maghemite (γ-Fe_2_O_3_) and ε-Fe_2_O_3_ [7,8,9].

On the other hand, hollow spherical nanostructures have gained significant attraction due to their unique architecture, which gives them low density, so they can float in different solutions, and significant interior spaces, which can enable their storage of different substances for use as photocatalysts, gas sensors, energy storage and chemotherapeutic drug delivery, among others [10,11,12,13,14,15]. Hollow iron oxide spheres have been synthesized using chemical and physical methods, which can produce a high size variability, defects and agglomeration, thus, reducing the effectiveness of their magnetic properties. These include solvothermal synthesis [16,17,18,19], the sol–gel method with a one-step reaction [20], a nanoscale Kirkendall diffusion process [21], and the template method [1,6,22]; however, the shape-controlled synthesis of hollow iron oxide nanospheres is still quite a challenge.

In this work, the atomic layer deposition (ALD) technique was combined with electrospinning to obtain novel hollow iron oxide nanospheres. In this case, the electrospinning technique was applied to produce polymeric spherical particles by applying a high voltage that broke the surface tension of the droplets of a polymeric solution, responsible for the size and shape of the particles, located at the tip of a needle [23,24]. Then, these polymeric spherical particles were coated using the ALD technique with a thin layer of Al_2_O_3_ that allowed these particles to be subjected to elevated temperatures. On this protective layer, a layer of Fe_2_O_3_ was deposited, obtaining spherical particles of PVP/Al_2_O_3_/Fe_2_O_3_. Finally, through a thermal reduction process carried out under a hydrogen atmosphere, the polymer disintegrated, and the hematite (Fe_2_O_3_) was transformed into magnetite (Fe_3_O_4_), thus, obtaining hollow Fe_3_O_4_ nanospheres. This is the first report on the development of hollow Fe_3_O_4_ nanospheres synthesized using both electrospinning and ALD techniques, presenting nanospheres that were fully characterized by measuring the morphological, structural and magnetic properties.

## 2. Experimental Details

### 2.1. Development of Hollow Fe_3_O_4_ Nanospheres

Novel hollow iron oxide nanospheres were successfully obtained following a four-step procedure:

#### 2.1.1. Electrospraying Process of PVP Spherical Particles

First, electrosprayed spherical poly(vinylpyrrolidone) (Sigma Aldrich, Santiago, Chile) (PVP) particles were obtained through a vertical electrospinning system (Spraybase^®^ power supply unit, Maytnooth, Ireland). In total, 2 g of poly(vinylpyrrolidone) was added to 10 mL of an ethanolic solution at 50% (*v*/*v*), and stirred at room temperature until the PVP was fully dissolved. The polymeric solution was collected in a 5 mL plastic syringe and connected through a polytetrafluoroethylene tube to a 0.9 mm diameter stainless-steel needle charged with a high-voltage power supply in the range of 0–20 kV. The PVP solution was electrosprayed by using a distance of 12 cm, a flow rate of 0.5 mL h^−1^ and a voltage of 12.9 kV for 1 h, in order to obtain spherical particles.

#### 2.1.2. Synthesis of Spherical PVP/Al_2_O_3_ Particles

Initially, the deposition of Al_2_O_3_ (50 cycles) using the Savannah S100 ALD reactor from Cambridge Nanotech (Cambridge, MA, USA) was necessary, supplying the role of maintaining the spherical shape of the PVP particles and avoiding their detachment during the Fe_2_O_3_ ALD step, which was carried out at a higher temperature [25]. Each cycle consisted of a 0.015 s pulse of trimethylaluminum (Al(CH_3_)_3_) (TMA) (Sigma Aldrich, Santiago, Chile) with a 30 s purge, followed by a 0.015 s pulse of ultrapure water (H_2_O) with a 60 s purge. The precursors were used at room temperature, while the reactor was kept at a temperature of 80 °C, thus, obtaining spherical PVP/Al_2_O_3_ particles.

#### 2.1.3. Synthesis of Spherical PVP/Al_2_O_3_/Fe_2_O_3_ Particles

Subsequently, the spherical PVP/Al_2_O_3_ particles were coated with 500 cycles of Fe_2_O_3_ in the ALD reactor at 200 °C in stop/exposure mode. The precursors used were ferrocene (FeCp_2_) and ozone (O_3_). The first was kept in a stainless-steel bottle, which was heated to 80 °C to ensure sufficient vapor pressure. On the other hand, the ozone at a volume concentration close to 10% was obtained from an ozone generator (Ol80W/FM100V) (Black Diamond, AB, Canada). The pulse times of ferrocene and ozone in the FeCp_2_/O_3_ cycle were 2 s and 0.2 s, respectively; the exposure and pump times were 5 s and 15 s, respectively, thus, obtaining spherical PVP/Al_2_O_3_/Fe_2_O_3_ particles [26]. It is important to note that throughout the process, a flow of 20 sccm of nitrogen was maintained.

#### 2.1.4. Thermal Reduction Process to Obtain Hollow Fe_3_O_4_ Nanospheres

**Figure 1:**
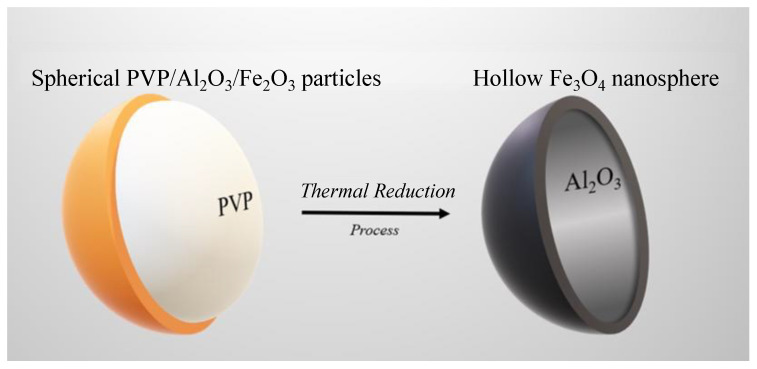
Scheme of the thermal reduction process that produced the calcination of the polymer and the transformation of hematite (Fe_2_O_3_) into magnetite (Fe_3_O_4_).

Finally, the spherical PVP/Al_2_O_3_/Fe_2_O_3_ particles were subjected to a thermal reduction process for which the sample was introduced into a GSL-1100X oven (Richmond, CA, USA) at 430 °C for 4 h under an atmosphere of hydrogen (4%) balanced with argon (96%) [27]. The temperature was chosen based on the results found by Espejo et al. [28], who pointed out that at approximately 430 °C, the peaks related to the hematite phase (Fe_2_O_3_) vanished and only the magnetite phase (Fe_3_O_4_) could be observed in the sample. This process caused two simultaneous situations: first, the calcination of the PVP polymer occurred, causing the spherical particles to become hollow spheres; second, hematite (Fe_2_O_3_) became magnetite (Fe_3_O_4_), resulting in hollow Fe_3_O_4_ nanospheres. The scheme in Figure 1 shows the thermal reduction process that produced the calcination of the polymer and the transformation of hematite (Fe_2_O_3_) into magnetite (Fe_3_O_4_).

### 2.2. Characterization Techniques

The morphology of the samples, before and after the thermal reduction process, was examined using a scanning electron microscope (Zeiss EVO MA10 SEM, Oberkochen, Germany) and a transmission electron microscope used at 120 kV (Hitachi HT7700 high-resolution TEM, Chiyoda, Tokyo, Japan). The images were recorded at different magnifications.

The thickness of the different coatings deposited by the ALD technique was measured indirectly using an alpha-SE ellipsometer from J. A. Wollam (Lincoln, NE, USA). In total, 50 cycles of Al_2_O_3_ and 500 cycles of Fe_2_O_3_ were deposited on Si(100) substrates, with 200 nm of thermally grown SiO_2_ (Ted Pella Inc., Redding, CA, USA).

The identification of the phases was carried out by means of X-ray diffraction (XRD) using a Shimadzu 6000 X-ray diffractometer (Nakagyo, Kyoto, Japan) (Cu-Kα radiation, 40 kV and 30 mA). All scans were performed at room temperature using a 2θ range of 10–70°.

All magnetic measurements were performed in a mini Cryogen Free System (mCFMS) vibrating sample magnetometer (VSM) from Cryogenic (London, W3 7QE, UK). The hysteresis curves were measured with a field between 4 and −4 kOe in the temperature range from 10 to 300 K.

## 3. Results and Discussion

### 3.1. Macroscopical Characterization

**Figure 2:**
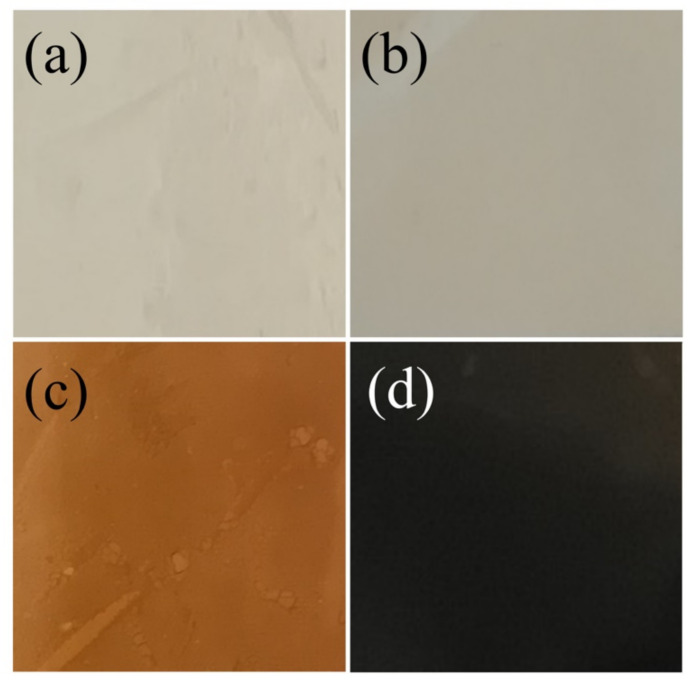
Photographs of: (**a**) electrosprayed spherical PVP particles, (**b**) deposition of 50 cycles of Al_2_O_3_ on spherical PVP particles with ALD (PVP/Al_2_O_3_), (**c**) deposition of 500 cycles of Fe_2_O_3_ on spherical PVP/Al_2_O_3_ particles with ALD (PVP/Al_2_O_3_/Fe_2_O_3_) and (**d**) thermal reduction of PVP/Al_2_O_3_/Fe_2_O_3_ particles to obtain hollow Fe_3_O_4_ nanospheres.

Figure 2a–d shows photographs of samples obtained through the four-step procedure, resulting in electrosprayed PVP solution, alumina-coated, alumina–hematite double-coated and magnetite particles, respectively. The alumina deposition (Figure 2b) maintained the initial white color of the collected electrosprayed spheres (Figure 2a), while the Fe_2_O_3_ deposition acquired a red color (Figure 2c). This change was related to the hematite presence, whose main absorption in the visible region was attributed to the charge transfer transition from the O 2p to Fe 3d levels, resulting in a red color [29]. In addition, the color tone of hematite powder strongly depends on its particle size and dispersibility, where a reddish color indicates that the particles are small and well dispersed [30]. Finally, the thermal reduction process produced a new color change in the sample (Figure 2d). The change in the crystalline structure was responsible for the observed black color due to the fact that Fe(II) and Fe(III) were found in octahedral sites in the magnetite structure that resulted in an intervalence charge transfer that rose to an absorption band at the second NIR region at 1000–1350 nm [31,32].

### 3.2. Morphological Characterization

**Figure 3:**
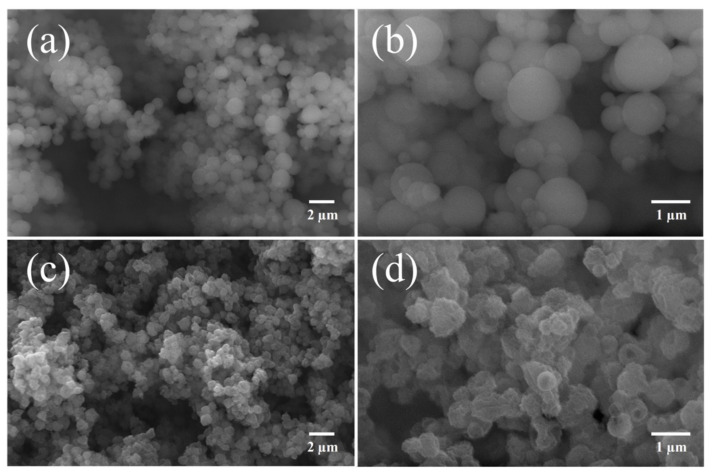
Scanning electron microscopy (SEM) images of (**a**,**b**) spherical PVP/Al_2_O_3_/Fe_2_O_3_ particles and (**c**,**d**) hollow Fe_3_O_4_ nanospheres.

The growth rate of alumina (Al_2_O_3_) was 0.05 nm/cycle, while that of hematite (Fe_2_O_3_) was 0.03 nm/cycle. These values were quite similar to those reported in the literature [33,34,35]. SEM images presented in Figure 3a,b revealed that the morphology of the PVP spheres was maintained after the deposition of alumina (PVP/Al_2_O_3_) and hematite (PVP/Al_2_O_3_/Fe_2_O_3_), ensuring uniformity and homogeneity. In addition, the above confirmed that the coating of the PVP spheres with a thin film of Al_2_O_3_ prevented the detachment of the substrate and the deterioration of the polymeric material.

On the other hand, the SEM images presented in Figure 3c,d show that the particles that were subjected to a thermal reduction process slightly changed their morphology, and although they maintained their spherical shape, it was less defined and rougher. This was due to two reasons: first, the PVP polymer inside the sphere, when heated, decomposed rapidly, generating volatile components that caused the breakage of the walls of the sphere due to the pressure of the gases inside [25,36]; second, the transformation of the coating from Fe_2_O_3_ to Fe_3_O_4_ implied that oxygen escaped through the coating, causing cracks in the walls of the hollow sphere [37]. The hollow Fe_3_O_4_ nanospheres ultimately resembled punctured ping-pong balls.

**Figure 4:**
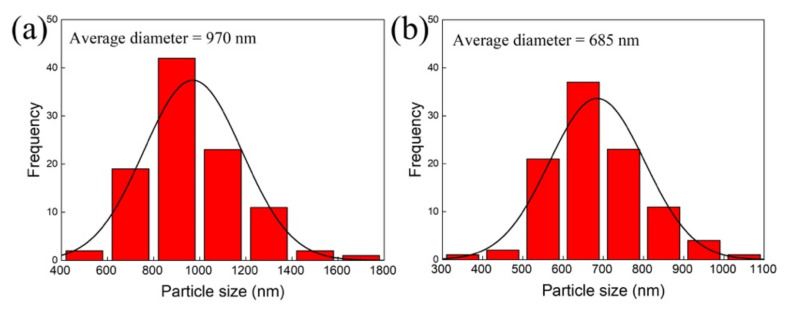
Size distribution of (**a**) spherical PVP/Al_2_O_3_/Fe_2_O_3_ particles and (**b**) hollow Fe_3_O_4_ nanospheres. The distribution used to measure the particle size corresponded to a normal distribution.

Figure 4a shows the size distribution of the spherical PVP/Al_2_O_3_/Fe_2_O_3_ particles (Figure 3a), while Figure 4b shows the hollow Fe_3_O_4_ nanospheres (Figure 3c). In both cases, ImageJ software (version 1.37, Bethesda, MD, USA) was used to obtain histograms with 100 particles. While spherical PVP/Al_2_O_3_/Fe_2_O_3_ particles exhibited an average diameter of 970 ± 213 nm, hollow Fe_3_O_4_ nanospheres exhibited a much smaller average diameter of 685 ± 119 nm. This almost 300 nm reduction in particle diameter was consistent with the fact noted above, that the thermal reduction process induced the formation of holes in the walls of the spheres.

**Figure 5:**
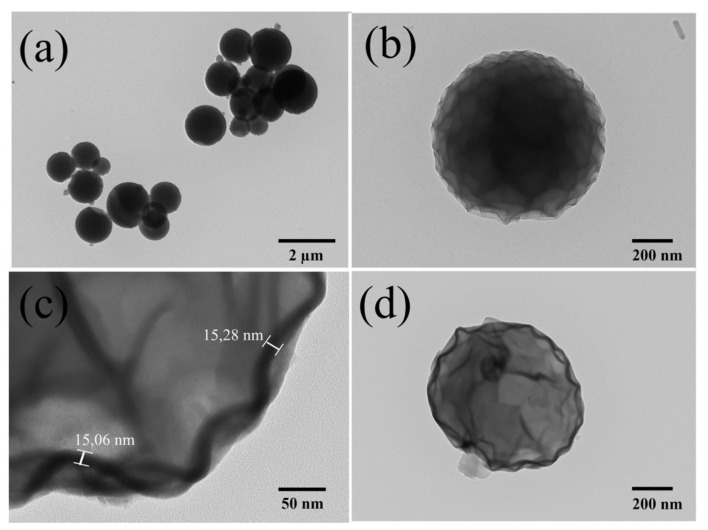
Transmission electron microscopy (TEM) images of (**a**,**b**) spherical PVP/Al_2_O_3_/Fe_2_O_3_ particles and (**c**,**d**) hollow Fe_3_O_4_ nanospheres.

The samples were also observed with transmission electron microscopy (TEM). While Figure 5a,b show the spherical PVP/Al_2_O_3_/Fe_2_O_3_ particles, Figure 5c,d show the hollow Fe_3_O_4_ nanospheres. By comparing Figure 5b,d, it is possible to observe the morphological change of a particle before and after the thermal reduction process, respectively. The spherical PVP/Al_2_O_3_/Fe_2_O_3_ particle presented a more regular surface compared to that exhibited by the hollow Fe_3_O_4_ nanosphere, on which we could also observe a contraction denoted mainly by the folds that were observed on the surface of its shell. Furthermore, it was possible to determine that the thickness of the material deposited with ALD was approximately 15 nm, a value that was obtained by measuring the difference in contrast observed in Figure 5c. This value agreed with the 15.27 ± 0.75 nm measured using ellipsometry when 500 cycles of Fe_2_O_3_ were deposited on a Si(100) substrate.

### 3.3. Structural Characterization

**Figure 6:**
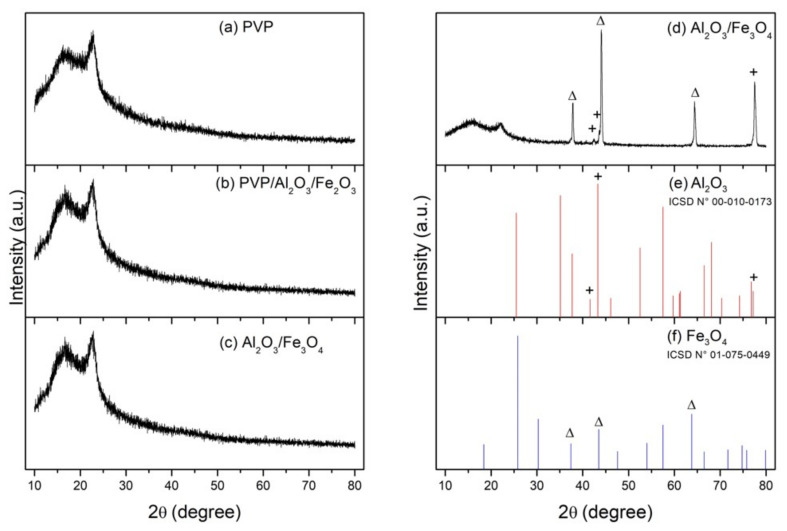
XRD patterns of (**a**) electrosprayed spherical PVP particles, (**b**) deposition of 50 cycles of Al_2_O_3_ on spherical PVP particles with ALD (PVP/Al_2_O_3_), (**c**) deposition of 500 cycles of Fe_2_O_3_ on spherical PVP/Al_2_O_3_ particles with ALD (PVP/Al_2_O_3_/Fe_2_O_3_), (**d**) thermal reduction of PVP/Al_2_O_3_/Fe_2_O_3_ particles to obtain hollow Fe_3_O_4_ nanospheres (+) and traces of Al_2_O_3_ (Δ), (**e**) diffraction peaks of standard card ICSD no. 01-010-0173 and (**f**) diffraction peaks of standard card ICSD no. 01-075-0449.

The X-ray diffraction (XRD) analysis of the samples in each of the four stages of the synthesis of the hollow Fe_3_O_4_ nanospheres is shown in Figure 6. The diffraction pattern of the PVP spheres showed a broad hump observed for values between 10 and 25° of 2θ, characteristic of the amorphous phase of the PVP polymer [38,39]. It was interesting to note that the diffraction patterns were not capable of detecting peaks associated with the materials deposited with ALD. This could be explained because the deposition process at low temperatures (below 500 °C) by means of ALD can produce coatings with a certain level of amorphousness [40]. Considering that the alumina (Al_2_O_3_) was deposited at 80 °C and that the hematite (Fe_2_O_3_) was deposited at 200 °C, the amorphous nature of these coatings evidenced in the diffraction patterns was expected.

For the sample obtained after the thermal reduction process, corresponding to the hollow nanospheres, the diffraction pattern was clearly different. First, the contribution of the PVP spheres was markedly reduced, because the temperature degraded the polymer. Second, the peaks (+) observed at 42.5°, 43.6° and 77.5° corresponded to the (006), (113) and (119) planes of alumina (Al_2_O_3_), which had a rhombohedral structure with lattice parameters a = b = 4.75 and c = 12.99 Å, belonging to the space group R-3c, according to the ICSD sheet no. 01-010-0173. Finally, the peaks (Δ) observed at 37.6°, 43.8° and 63.9° corresponded to the (222), (400) and (440) planes of magnetite (Fe_3_O_4_), which had a cubic structure with lattice parameters a = b = c = 8.31 Å, belonging to the space group Fd-3m, according to the ICSD sheet no. 01-075-0449.

### 3.4. Magnetic Characterization of Hollow Fe_3_O_4_ Nanospheres

The measured sample corresponded to a powder containing hollow Fe_3_O_4_ nanospheres exhibiting an average diameter of 685 ± 119 nm (see Figure 4b). The thickness of alumina (Al_2_O_3_) measured using ellipsometry was 2.48 ± 0.05 nm, while 500 cycles of hematite (Fe_2_O_3_) produced a thickness of 15.27 ± 0.75 nm. This meant that the total thickness of the material deposited with ALD (Al_2_O_3_/Fe_2_O_3_) was approximately 18 nm. However, after the thermal reduction process, to transform hematite (Fe_2_O_3_) into magnetite (Fe_3_O_4_), a contraction of approximately 3 nm was produced due to the dewetting process [41], where the release of oxygen produced cracks or holes in the surface, thus reducing, the thickness of the deposited material to approximately 15 nm (see Figure 5c).

**Figure 7:**
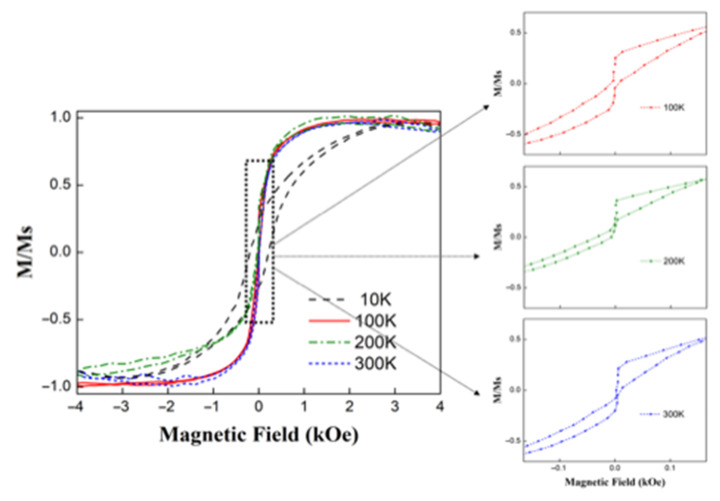
Hysteresis curves for hollow Fe_3_O_4_ nanospheres obtained at different temperatures.

Figure 7 shows the hysteresis loops for hollow Fe_3_O_4_ nanospheres obtained at different temperatures. It is possible to observe that the hysteresis curve for a temperature of 10 K was much squarer than the other hysteresis curves, and exhibited a much higher coercivity. The hysteresis curves obtained for temperatures ranging between 100 and 300 K were very similar to each other, which ensured that the magnetic properties of these hollow nanospheres did not vary much in this temperature range when they were used in different applications. It was interesting to note that the remanence took generally small values at all temperatures, so the contribution from magnetostatic interactions may have been relevant. Additionally, there was a diamagnetism associated with the alumina (Al_2_O_3_) coating that increased with temperature.

**Figure 8:**
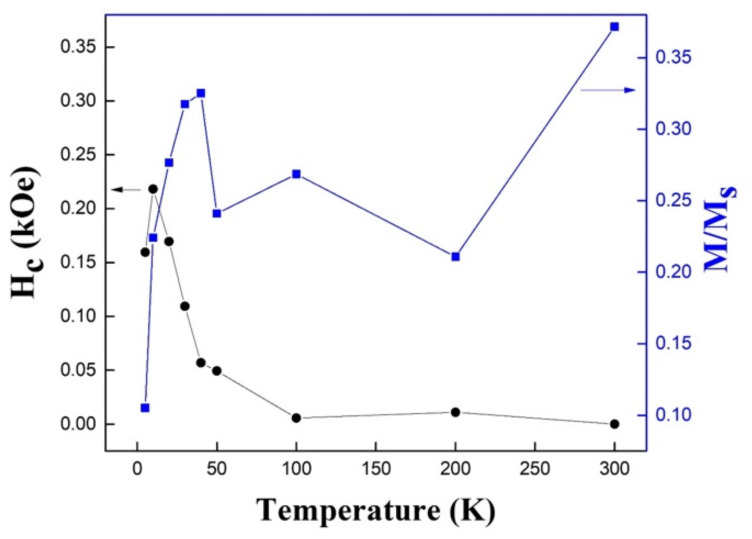
Coercivity (black circles) and reduced remanence (blue squares) for hollow Fe_3_O_4_ nanospheres as a function of temperature.

In general, magnetite (Fe_3_O_4_) nanoparticles exhibit a superparamagnetic behavior, such as those synthesized by the coprecipitation of iron (II) and iron (III) with sodium hydroxide in an aqueous solution at high temperature (80 °C), which have an average diameter of 15 nm [42]. In Figure 8, the coercivity and reduced remanence were summarized as a function of the temperature of the hollow Fe_3_O_4_ nanospheres. From this figure, we could conclude that the coercivity varied between approximately 0.0 and 0.22 kOe, while the reduced remanence varied between 0.02 and 0.37. The coercivity presented a nonmonotonic behavior, since it began to increase until reaching a maximum value close to 20 K, a temperature from which the coercivity presented a sustained decrease to reach an anhysteretic behavior (almost zero coercivity and remanence) for 100 K. A higher temperature would not result in significant changes in coercivity. Similarly, the reduced remanence also presented a nonmonotonic behavior, reaching a peak at a temperature close to 40 K, after which it fell to a valley that remained approximately constant until 200 K, after which the temperature increased again to reach the maximum at 300 K. The fact of the obtainment of low coercivities and remanence for a wide range of temperatures could be useful in applications where the agglomeration of magnetic nanostructures in liquid media is not allowed, such as drug encapsulation and delivery.

## 4. Conclusions

This was the first report on the development of hollow iron oxide nanospheres obtained from a combination of electrospinning and atomic layer deposition techniques. Although the purpose of this article was to obtain hollow magnetic spheres, it was first necessary to coat the polymeric (PVP) spheres with a thin layer of alumina (Al_2_O_3_). A layer of approximately 15 nm of hematite (Fe_2_O_3_) was then deposited on these spheres, a process that maintained the spherical shape of the particles. Finally, the spheres were subjected to a thermal reduction process that allowed the obtainment of the hollow Fe_3_O_4_ nanospheres. The fact that they were hollow was due to the temperature being sufficient enough to degrade the polymer (PVP) that was inside the spheres, and the presence of a hydrogen atmosphere facilitated the transformation of most of the hematite (Fe_2_O_3_) into magnetite (Fe_3_O_4_). It is important to point out that these processes produced a contraction in the size of the particles, while their surface became much more irregular. The hysteresis curves of the hollow iron oxide nanospheres allowed us to observe low coercivities and reduced remanence in a wide range of temperatures, allowing us to suggest their use in applications where the agglomeration of magnetic nanostructures in liquid media is not allowed, such as drug encapsulation and delivery. This four-step synthesis process could be used to obtain hollow nanospheres from other ferromagnetic materials and their alloys.

## Data Availability

Data are available on request.
